# Patient safety management systems and activities related to promoting voluntary in-hospital reporting and mandatory national-level reporting for patient safety issues: A cross-sectional study

**DOI:** 10.1371/journal.pone.0255329

**Published:** 2021-07-28

**Authors:** Shigeru Fujita, Kanako Seto, Yosuke Hatakeyama, Ryo Onishi, Kunichika Matsumoto, Yoji Nagai, Shuhei Iida, Tomohiro Hirao, Junko Ayuzawa, Yoshiko Shimamori, Tomonori Hasegawa

**Affiliations:** 1 Toho University School of Medicine, Tokyo, Japan; 2 Hitachinaka General Hospital, Ibaraki, Japan; 3 Nerima General Hospital, Tokyo, Japan; 4 Institute for Healthcare Quality Improvement, Tokyo, Japan; 5 Faculty of Medicine, Kagawa University, Kagawa, Japan; 6 Faculty of Medical Science, Kyushu University, Fukuoka, Japan; 7 Iwate Medical University School of Nursing, Iwate, Japan; Hong Kong Polytechnic University, HONG KONG

## Abstract

Both voluntary in-hospital reporting and mandatory national-level reporting systems for patient safety issues need to work well to develop a patient safety learning system that is effective in preventing the recurrence of adverse events. Some of the hospital systems and activities may increase voluntary in-hospital reporting and mandatory national-level reporting. This study aimed to identify the hospital systems and activities that increase voluntary in-hospital reporting and mandatory national-level reporting for patient safety issues. An anonymous mail survey of hospitals in Japan was conducted in 2017. The hospitals were selected by stratified random sampling according to number of beds. The survey examined the annual number of reported events in the voluntary in-hospital reporting system for patient safety and experience of reporting unexpected patient deaths possibly due to medical interventions to the mandatory national-level reporting system in the last 2 years. The relationship of the answer to the questions with the patient safety management systems and activities at each hospital was analyzed. The response rate was 18.8% (603/3,215). The number of in-hospital reports per bed was positively related to identifying events by referring complaints or questions of patients or family members, using root cause analysis for analyzing reported events, and developing manuals or case studies based on reported events, and negatively related to the unification and standardization of medical devices and equipment. The experience with mandatory national-level reporting of serious adverse events was positively related to identifying problematic cases by a person in charge of patient safety management from the in-hospital reporting system of complications and accidental symptoms. Enhanced feedback for reporters may promote voluntary in-hospital reporting of minor cases with low litigation risks. Developing an in-hospital mechanism that examines all serious complications and accidental symptoms may promote mandatory national-level reporting of serious adverse events with high litigation risks.

## Introduction

A reporting culture is one of the significant components of patient safety culture [1]. Although patient safety learning systems may not function well due to underreporting, they have been constructed at hospital and national levels in several countries [1–6]. The patient safety learning system consists of adverse event and near miss reporting, investigation, analysis, and feedback; the proactive reporting by a person involved in the error is a significant foundation of the system [4]. In the patient safety learning system, healthcare workers are encouraged to report near misses, adverse events or sentinel events to in-hospital reporting system and sometimes to a national-level reporting system [2, 5, 6]. Several studies have reported barriers to in-hospital reporting, including fear of blame, insufficient feedback to reporters, lack of organizational support, and the perception that reporting does not result in improvement to patient safety [3–8]. Conversely, previous studies have reported the drivers, including a shorter time to report, a trigger list to help healthcare workers understand what to report, providing enhanced feedback on errors and hazards, anonymous reporting, assignment of full-time patient safety managers, education, and training [4–9]. As for the feedback to healthcare workers and reporters, hospitals are encouraged to provide regular feedback on statistics of recently reported events or hazard control strategies found from recent analysis, but more means of feedback may be needed to promote reporting [4, 10]. Knowledge obtained from serious adverse events with low incidence may need to be widely shared to give other hospitals an opportunity to prevent uncommon serious adverse events. Several countries, including France, Italy, Norway and Mexico, have developed a national-level reporting system, which collects adverse events and/or near misses from hospitals [1, 2]. The barriers to national-level reporting, include a lack of funding for the system, fear of sanctions, less involvement of physicians, and few participating hospitals [1]. In the United Kingdom, although the under-reporting of serious adverse events was considered a challenge, a voluntary reporting system of adverse events at the national level was developed in 2004, and the English government introduced a mandatory reporting system for serious adverse events with penalties for delayed reporting in 2010 [11]. Reducing the under-reporting of serious adverse events may be challenging despite the introduction of mandatory reporting system because some serious adverse events may be deliberately considered by a person involved in the error as a complication that does not need to be reported [12]. The mandatory national-level reporting system for serious adverse events may have different drivers of reporting compared with the voluntary in-hospital reporting system. The introduction of the immunity system for reporters is one of the means to promote mandatory reporting; however, it can involve various challenges, such as securing a budget and obtaining a consensus from funders of the budget [2, 11]. Other drivers for mandatory national-level reporting that can be achieved in each hospital need to be identified.

In Japan, hospitals are requested to have a voluntary in-hospital reporting system for adverse events and near misses; however, the number of reports depends on various factors and varies among hospitals [9]. Additionally, the hospitals are requested to report unexpected patient deaths possibly due to medical interventions to a third-party organization named the Medical Accident Investigation and Support Center since 2015 [2]. In 2019, 373 lethal adverse events were reported to the Center, and 41.8% of those reported cases were caused by surgeries [13]. Although there is no explicit penalty for not reporting to the Center, and differences in reporting attitudes among hospitals may exist, it is a duty of the hospital director to report cases that meet the criteria of reporting.

Both voluntary in-hospital reporting and mandatory national-level reporting systems need to work well to develop effective patient safety learning systems; however, to promote them seems to require room for improvement. More drivers of reporting may need to be identified from the various patient safety management activities and quality improvement initiatives in the hospitals. This study aimed to identify the hospital systems and activities that increase voluntary in-hospital reporting and mandatory national-level reporting on patient safety issues.

## Methods

The Ethics Committee of Toho University approved the study (A17025). An anonymous mail survey of hospitals in Japan was conducted in 2017.

### Subjects

The hospitals were selected by stratified random sampling according to number of beds: 25% of hospitals with <100 beds, 50% of hospitals with 100–299 beds, and 100% hospitals with ≥300 beds were selected. Consequently, a questionnaire was sent to 3,215 hospitals, representing 38% (3,215/8,448) of all hospitals in Japan. Respondents were hospital directors or persons in charge of patient safety management at the hospitals. This study was an exploratory study and did not predetermine the power and sample size.

### Questionnaire

In this study, we hypothesized that the number of voluntary in-hospital reports on adverse events and near misses and the mandatory national-level reports on serious adverse events would be related to the patient safety management system; the ways to collect, analyze, and utilize information of events occurring in the hospital and other initiatives for each hospital’s quality improvement. The survey included questions regarding hospital function, number of beds, hospital accreditation status, assignment of dedicated patient safety managers and healthcare mediators, methods to detect events occurring in hospitals, analysis method of reported events, how to use the information from reported events, and implemented initiatives for quality improvement (Table 1). Moreover, the survey asked each respondent regarding the annual number of reported events in the in-hospital reporting system for patient safety and if the hospital had reported unexpected patient deaths possibly due to medical interventions to the national-level reporting system in the last 2 years.

**Table 1:** The characteristics of respondents.

Characteristics		All subjects (n = 603)		Annual number of in-hospital reports per bed	Hospitals that have experience of national-level reports (n = 114)		Hospitals that have NO experience of national-level reports (n = 480)		Experience vs. NO experienceχ^2^
		n	(%)	Mean ± SD	n	(%)	n	(%)	P
Hospital function									
	Acute care hospitals	471	(78)	4.36 ± 3.42	110	(96)	353	(74)	<0.01
	Long-term care, psychiatric or other hospitals	132	(22)	2.92 ± 3.65	4	(4)	127	(26)	
No. of beds									
	<200 beds	267	(44)	3.61 ± 3.73	17	(15)	248	(52)	<0.01
	200–399 beds	181	(30)	4.10 ± 2.94	45	(39)	132	(28)	
	≥400 beds	152	(25)	4.77 ± 3.67	51	(45)	98	(20)	
	NA	3	(0)	-	1	(1)	2	(0)	-
Critical care center		91	(15)	4.86 ± 2.21	41	(36)	50	(10)	<0.01
Accreditation status									
	Accredited hospital	320	(53)	4.60 ± 3.65	88	(77)	226	(47)	<0.01
	Non-accredited hospital	283	(47)	3.40 ± 3.24	26	(23)	254	(53)	
Dedicated patient safety managers									
	Assigned	457	(76)	4.39 ± 3.44	110	(96)	341	(71)	<0.01
	Not assigned	146	(24)	2.92 ± 3.56	4	(4)	139	(29)	
Healthcare mediators									
	Assigned	301	(50)	4.22 ± 2.85	70	(61)	230	(48)	0.01
	Not assigned	291	(48)	3.88 ± 4.13	44	(39)	247	(51)	
	NA	11	(2)	-	0	(0)	3	(1)	-
Methods for a person in charge of patient safety management to discover events occurring in the hospital									
	Referring complaints or questions from patients or family members	486	(81)	4.28 ± 3.71	97	(85)	388	(81)	0.29
	Careful examination for all inpatient deaths	319	(53)	4.46 ± 3.61	92	(81)	227	(47)	<0.01
	In-hospital reporting system of complications and accidental symptoms	223	(37)	4.74 ± 3.70	70	(61)	152	(32)	<0.01
	Acquiring contents of clinical conference	128	(21)	4.00 ± 3.01	21	(18)	107	(22)	0.37
	Acquiring contents of morbidity & mortality conference	90	(15)	4.34 ± 2.66	27	(24)	63	(13)	<0.01
Analysis method to identify the cause of adverse events									
	No specific method is used	222	(37)	3.10 ± 2.58	30	(26)	188	(39)	<0.01
	Non–RCA method^¶^	174	(29)	4.10 ± 2.89	32	(28)	142	(30)	
	Root cause analysis (RCA)	202	(33)	5.01 ± 4.49	52	(46)	150	(31)	
	NA	5	(1)	-	0	(0)	0	(0)	-
How to use reported events									
	Education or training based on reported events	466	(77)	4.12 ± 3.40	94	(82)	370	(77)	0.21
	Regular aggregation of frequency or pattern of events	359	(60)	4.06 ± 3.35	72	(63)	287	(60)	0.51
	Developing manuals or case studies based on reported events	299	(50)	4.52 ± 4.04	67	(59)	232	(48)	<0.05
Implemented initiatives for quality improvement									
	Regular review of manuals & rules	499	(83)	3.99 ± 3.10	99	(87)	399	(83)	0.33
	Regular measurement of patient satisfaction	419	(69)	4.29 ± 3.46	93	(82)	325	(68)	<0.01
	Regular measurement of employee satisfaction	263	(44)	4.23 ± 2.97	61	(54)	201	(42)	0.02
	Unification and standardization of medical devices and equipment	242	(40)	3.90 ± 2.47	53	(46)	187	(39)	0.14
	Monitoring compliance with manuals & rules	181	(30)	4.57 ± 3.31	53	(46)	128	(27)	<0.01
	Regular measurement of patient safety & quality indicators	142	(24)	4.74 ± 2.95	40	(35)	102	(21)	<0.01
	Standardization of patient information handoff process	114	(19)	4.37 ± 2.37	35	(31)	79	(16)	<0.01
	Distributing JQ patient safety alerts to all staff individually^§^	82	(14)	4.13 ± 2.26	21	(18)	61	(13)	0.11
	Establishing a team to rapidly respond to worsening or sudden changes in patient condition	64	(11)	5.42 ± 3.33	18	(16)	46	(10)	0.05

¶: Analysis methods unique to Japan, such as the SHELL model, ImSAFER, or others

§: JQ: The Japan Council for Quality Health Care is a hospital accreditation organization in Japan.

The survey asked how patient safety alerts were used at each hospital. The Japan Council for Quality Health Care, which is an accreditation body, maintains a nationwide adverse event and near miss voluntary reporting system and issues the patient safety alerts monthly based on the reported events [14]. The alerts are generally used to educate healthcare workers in each hospital. The survey asked if the hospital assigned healthcare mediators. Establishing a hospital support system for the persons involved in an adverse event may remove a barrier to reporting [4]. In Japan, hospitals are reimbursed if there is a patient consultation service counter for patient safety issues and if healthcare mediators are assigned in the counter. The healthcare mediators are responsible for facilitating dialogs between healthcare professionals and patients and their families, especially in the event of a conflict. Most of them are nurses, clerks, and medical social workers [15]. The survey asked if the persons in charge of patient safety management receive voluntary in-hospital reports of complications and accidental symptoms and identify patient safety issues in them. The survey examined the analysis method used in each hospital to identify the cause of adverse events. In Japan, root cause analysis (RCA) is a popular analysis method of reported events, although some other methods that are not popular in other countries, such as the Software-Hardware-Environment-Liveware model or the Improvement for Medical System by Analyzing Fault Root in Human Error Incident (ImSAFER), are also used [16, 17]. In this study, those methods were categorized as non-RCA.

### Outcomes

The main outcomes in this study are the annual number of reported events per bed in the voluntary in-hospital reporting system for patient safety, and whether the hospital has reported unexpected patient deaths possibly due to medical interventions to the mandatory national-level reporting system in the last 2 years.

### Data analyses

The relationship of the outcomes with the patient safety management systems and activities at each hospital was analyzed by generalized linear models (GZLMs), with identity link function and logit link function for the annual number of in-hospital reports per bed and experience of reporting unexpected patient deaths, respectively. Missing data were excluded from the analysis. This study did not collect information about hospital region and did not apply a multi-level model. The Chi-square test was performed to compare categorical variables. The Student’s t-test was used to compare the mean annual number of in-hospital reports per bed. Statistical analyses were performed using the SPSS 25.0 (IBM, Armonk, NY), and P < 0.05 was used to determine significance.

## Results

The response rate was 18.8% (603/3,215). The characteristics of respondents are shown in Table 1. The annual number of in-hospital reports per bed was 4.05 ± 3.52 (mean ± standard deviation). Acute care hospitals had a higher annual number of in-hospital reports per bed than other hospitals (4.36 ± 3.42 vs. 2.92 ± 3.65, P < 0.01). Furthermore, hospitals with ≥200 beds had a higher annual number of in-hospital reports per bed than hospitals with <200 beds (4.40 ± 3.30 vs. 3.61 ± 3.73, P <0.01), and hospitals with critical care center had a higher annual number of in-hospital reports per bed than hospitals without it (4.86 ± 2.21 vs. 3.91 ± 3.69, P < 0.01). Unexpected patient deaths were reported to the mandatory national-level reporting system by 18.9% of the respondents. The proportion in acute care hospitals was higher than that in other hospitals (23.8% vs. 3.1%, P < 0.01). Moreover, the proportion in hospitals with ≥200 beds was higher than that in hospitals with <200 beds (29.4% vs. 6.4%, P < 0.01), and that in hospitals with a critical care center was higher than that in hospitals without one (45.1% vs. 14.4%, P < 0.01).

The GZLM results are shown in Table 2. The Akaike’s information criterion of GZLMs for the annual number of in-hospital reports per bed and the experience of national-level reports were 2954.9 and 506.6, respectively. The number of in-hospital reports per bed was positively related to identifying events by referring complaints or questions of patients or family members (β = 1.19, P < 0.01), using an RCA for the reported events analysis (β = 1.33, P < 0.01), and developing manuals or case studies based on the reported events (β = 0.70, P = 0.02), and negatively related to the unification and standardization of medical devices and equipment (β = −0.65, P = 0.03). The experience with the national-level reporting of serious adverse events was positively related to acute care hospital (OR = 3.89, P = 0.04), critical care center (OR = 2.69, P < 0.01), and identifying problematic cases by a person in charge of patient safety management from the voluntary in-hospital reporting system of complications and accidental symptoms (OR = 1.99, P < 0.01).

**Table 2:** The factors related to increased reporting.

		Annual number of in-hospital reports per bed^†^			Experience of national-level reports^††^			
		β	SE	P	β	SE	OR	P
Acute care hospital		0.64	0.41	0.12	1.36	0.65	3.89	0.04
No. of bed								
	<200 beds	reference			reference		1.00	
	200–399 beds	−0.10	0.37	0.78	0.80	0.36	2.23	0.02
	≥400 beds	0.03	0.49	0.95	0.39	0.44	1.47	0.37
Critical care center		0.19	0.50	0.71	0.99	0.35	2.69	<0.01
Accredited hospital		0.64	0.35	0.07	0.45	0.30	1.57	0.13
Assignment of dedicated patient safety managers		0.12	0.44	0.79	0.93	0.63	2.54	0.14
Assignment of healthcare mediators		−0.12	0.30	0.70	0.01	0.26	1.01	0.98
Methods for a person in charge of patient safety management to discover events occurring in the hospital								
	Referring complaints or questions from patients or family members	1.19	0.40	<0.01	−0.04	0.35	0.96	0.92
	Careful examination for all inpatient deaths	−0.01	0.34	0.98	0.58	0.31	1.79	0.06
	In-hospital reporting system of complications and accidental symptoms	0.60	0.33	0.07	0.69	0.26	1.99	<0.01
	Acquiring contents of clinical conference	−0.19	0.36	0.60	−0.53	0.32	0.59	0.10
	Acquiring contents of morbidity & mortality conference	0.04	0.43	0.93	0.28	0.31	1.32	0.38
Analysis method to identify the cause of adverse events								
	No specific method is used	reference			reference		1.00	
	Non–RCA method^¶^	0.61	0.37	0.10	−0.28	0.33	0.75	0.39
	Root cause analysis (RCA)	1.33	0.37	<0.01	0.16	0.30	1.17	0.61
How to use reported events								
	Education or training based on reported events	−0.23	0.36	0.53	−0.21	0.33	0.81	0.53
	Regular aggregation of frequency or pattern of events	−0.28	0.30	0.35	−0.13	0.26	0.88	0.63
	Developing manuals or case studies based on reported events	0.70	0.30	0.02	0.32	0.26	1.38	0.21
Implemented initiatives for quality improvement								
	Regular review of manuals & rules	−0.53	0.41	0.19	0.07	0.37	1.07	0.85
	Regular measurement of patient satisfaction	0.29	0.40	0.47	−0.19	0.36	0.83	0.61
	Regular measurement of employee satisfaction	−0.49	0.34	0.15	−0.19	0.28	0.83	0.50
	Unification and standardization of medical devices and equipment	−0.65	0.30	0.03	−0.12	0.26	0.88	0.63
	Monitoring compliance with manuals & rules	0.32	0.33	0.33	0.46	0.26	1.59	0.07
	Regular measurement of patient safety & quality indicators	0.13	0.37	0.74	−0.30	0.28	0.74	0.30
	Standardization of patient information handoff process	−0.42	0.40	0.29	0.02	0.29	1.02	0.95
	Distributing JQ patient safety alerts to all staff individually^§^	−0.42	0.42	0.31	0.12	0.32	1.12	0.71
	Establishing a team to rapidly respond to worsening or sudden changes in patient condition	0.91	0.50	0.07	−0.38	0.36	0.68	0.29

SE: Standard Error, OR: Odds Ratio

†: Estimation of generalized linear models with identity link function

††: Estimation of generalized linear models with logit link function

¶: Analysis methods unique to Japan, such as the SHELL model, ImSAFER, or others

§: JQ: The Japan Council for Quality Health Care is a hospital accreditation organization in Japan.

## Discussion

Larger acute care hospitals with critical care centers had more voluntary in-hospital reports and are more likely to have mandatory national-level report experiences than other hospitals; however, this may reflect the fact that patients with high medical needs are more likely to have adverse events or near misses. The hospital systems and activities associated with increased reporting were different between the voluntary in-hospital reporting and mandatory national-level reporting systems. The difference between them may owe to the difference in the characteristics of the reported cases. Near misses and serious adverse events are widely reported to the voluntary in-hospital reporting system by healthcare workers, although most of them are only minor cases with low litigation risks [14]. On the other hand, the mandatory national-level reporting system deals with only lethal cases with high litigation risks [13]. If the outcome of the case is not serious, the hospital activities that may lead to quality improvement, such as using an RCA for analyzing reported events and developing manuals or case studies based on reported events, may constitute positive feedback to the reporters, and the reporting may be regarded as a successful experience. Furthermore, previous studies suggest that an enhanced feedback process may increase voluntary in-hospital reporting [3–11]. However, in the case of serious outcomes, the reporter may not be able to expect positive feedback and may be less willing to report them. In addition, it is challenging to clearly distinguish complications from adverse events due to medical interventions, resulting in the difference in reporting standards among healthcare workers [12, 18, 19]. This suggests that some of the cases considered as complications may include serious adverse events that should be reported to the mandatory national-level reporting system. In the case of serious outcomes, the number of mandatory national-level reporting may be increased by establishing a system to discover both serious adverse events and complications that occurred in the hospital.

According to the GZLM results, the number of voluntary in-hospital reporting for patient safety may be increased by better feedback processes such as using patient’s complaints or questions to do with patient safety issues, using an RCA, and using reported events in a visible manner, such as developing manuals based on them. Patients’ complaints or opinions on healthcare services may include safety issues and should be communicated to the person in charge of patient safety management at the hospital. This feedbacks may help healthcare workers recognize the necessity of further reporting. Previous studies have suggested the positive effects of an RCA on the number of in-hospital reports and patient safety culture with no blame, suggesting that an RCA may have some positive secondary effects on patient safety [20, 21]. The RCA participants are expected to identify system errors behind human errors and improve work processes [16]. When healthcare workers participate in an RCA team, they may become aware that adverse events are caused by multiple organizational failures, which may lead to focusing on identifying system errors [21]. Therefore, a no-blame culture may be fostered and the in-hospital reporting may be promoted. Feedback to the reporters of in-hospital reports is significant in maintaining increased reporting [4, 10, 22]. Creating manuals or case studies based on the reported events may provide clear feedback to the reporters, and those who feel that their experiences and opinions have been utilized may then continue to report in the future. The unification and standardization of medical devices and equipment may not be a driver of reporting, but it may reduce adverse events and near misses.

According to the GZLM results, the experience with the national-level reporting of serious adverse events was related to only one hospital activity: identifying problematic cases by a person in charge of patient safety management from the voluntary in-hospital reporting system of complications and accidental symptoms. Serious complications and accidental symptoms, wherein the healthcare workers do not feel the need to be reported as patient safety issues, may include some unexpected patient deaths possibly due to medical interventions. Implementing a system that allows the person in charge of patient safety management to review serious complications may improve healthcare workers’ sensitivity to problematic cases and complications to be reported and raise awareness among healthcare workers regarding a need for transparency and accountability for those cases. In addition, the introduction of anonymous reporting and no-fault compensation schemes may increase the reporting of serious adverse events by health care providers because cases with high litigation risks are more likely to be underreported [8, 23, 24]. The previously reported barriers of national-level reporting, such as fear of sanctions or insufficient knowledge of what to report, also need to be improved [1].

There could be limitations regarding the representativeness of the respondents in this study because the valid response represented only 7.1% (603/8,448) of all hospitals in Japan. The number of in-hospital reports and the experience of national-level reporting can be overestimated because the hospitals with a good reporting culture may have been more likely to have responded to our survey. We believe that an increase in the number of in-hospital reports means an improvement in the reporting culture, but it may also reflect an increase in adverse events and near misses. The impact of increased reporting on patient safety outcomes, such as mortality ratio or the reduction of serious adverse events, needs to be evaluated in the future.

## Conclusions

The number of voluntary in-hospital reporting of adverse events and near misses was associated with using patients’ complaints or questions about patient safety issues, the use of an RCA, and developing manuals and case studies based on reported events. Enhanced feedback for reporters may promote voluntary in-hospital reporting of minor cases with low litigation risks. The experience with mandatory national-level reporting of serious adverse events was associated with implementing a system allowing the person in charge of patient safety management to review serious complications and accidental symptoms. Developing an in-hospital mechanism that examines all serious complications and accidental symptoms may promote mandatory national-level reporting of serious adverse events with high litigation risks.
